# Internet-based treatment for PTSD reduces distress and facilitates the development of a strong therapeutic alliance: a randomized controlled clinical trial

**DOI:** 10.1186/1471-244X-7-13

**Published:** 2007-04-19

**Authors:** Christine Knaevelsrud, Andreas Maercker

**Affiliations:** 1 Treatment Center for Torture Victims, Berlin, Germany; 2 University of Zurich, Dept. of Psychopathology and Clinical Intervention, Zürich, Switzerland

## Abstract

**Background:**

The present study was designed to evaluate the efficacy of an internet-based therapy (Interapy) for Posttraumatic Stress Disorder (PTSD) in a German speaking population. Also, the quality of the online therapeutic relationship, its development and its relevance as potential moderator of the treatment effects was investigated.

**Method:**

Ninety-six patients with posttraumatic stress reactions were allocated at random to ten sessions of Internet-based cognitive behavioural therapy (CBT) conducted over a 5-week period or a waiting list control group. Severity of PTSD was the primary outcome. Secondary outcome variables were depression, anxiety, dissociation and physical health. Follow-up assessments were conducted at the end of treatment and 3 months after treatment.

**Results:**

From baseline to post-treatment assessment, PTSD severity and other psychopathological symptoms were significantly improved for the treatment group (intent-to-treat group × time interaction effect size *d *= 1.40). Additionally, patients of the treatment condition showed significantly greater reduction of co-morbid depression and anxiety as compared to the waiting list condition. These effects were sustained during the 3-months follow-up period. High ratings of the therapeutic alliance and low drop-out rates indicated that a positive and stable therapeutic relationship could be established online. Significant improvement of the online working alliance in the course of treatment and a substantial correlation between the quality of the online relationship at the end of treatment and treatment outcome emerged.

**Conclusion:**

Interapy proved to be a viable treatment alternative for PTSD with large effect sizes and sustained treatment effects. A stable and positive online therapeutic relationship can be established through the Internet which improved during the treatment process.

**Trial registration:**

Australian Clinical Trials Registry ACTRN012606000401550

## Background

On the Internet there are thousands of virtual communities that specifically involve issues of substantial personal significance, among them sexual violence, child abuse, loss and, grief, as well as suicide. Typically, these websites provide information and offer forums to share and discuss these experiences. A considerable number of trauma victims use this medium as a way of coping with their experiences. Traumatic experiences are often associated with stigmatization and intense feelings of shame and guilt [1]. In addition, many victims report feeling alienated and estranged from the world. They refrain from social interactions and feel isolated although at the same time they often experience a great need for social support [2,3]. The Internet provides a protected environment where participants can easily control and regulate the degree of intimacy they want to share without the fear of real-life judgment, rejection, or devaluation. This way of communication lessens social risks and inhibitions and encourages the disclosure of painful experiences or shameful thoughts [4-6]. Van de Werker and Prigerson [7] were among the first researchers to provide evidence on the protective effect of Internet use and email contact in bereaved individuals (N = 293). They explored the amount of Internet communication post loss at different time points and found that the use of the Internet served as a protection against psychiatric illness secondary to bereavement and that it also enhanced quality of life.

The therapeutic community has only recently discovered the therapeutic potential that the Internet offers [for a review see [8]]. Lange et al. [9] were developing a pioneering Internet based therapy for trauma victims by combining a manual-based cognitive-behavioural writing therapy with the Internet (Interapy). As several face-to-face trials have proven, cognitive-behavioural therapy (CBT) is a powerful and effective method of treating posttraumatic stress disorder (PTSD) [10]. Lange et al. [9] showed that CBT could be successfully applied to the Internet. In a random controlled trial they treated 101 patients with posttraumatic stress (PTS). They showed that participants of the treatment group experienced a significant PTS symptom reduction and improvement in other psychopathological symptoms as compared to participants in the waiting list condition. This is the first study in another language which aims to replicate the results of Lange et al. [9] and thereby to validate this treatment approach cross-culturally.

Furthermore, we were interested in exploring the development and relevance of the online therapeutic relationship. While the treatment rationale of Interapy closely resembles conventional face-to-face CBT approaches with regard to content, the mode of delivery is fundamentally different. In face-to-face treatment therapists and patients see each other, share the same physical space, and are engaged in synchronous verbal and nonverbal communication. Online therapy is based on written (asynchronous) communication, geographical distance and visual anonymity. The therapeutic alliance also known as "working alliance" or "helping alliance" is conceived as an agreement on therapeutic goals and therapeutic tasks. It is also an agreement about the development of bonds of mutual trust, acceptance, and confidence between patient and therapist [11,12]. The quality of the therapeutic relationship has been found to be important to the outcome in different forms of face-to-face therapy [for meta-analysis see [13,14]]. Until now, very few empirical studies focused on the relevance of the therapeutic relationship online. Cook and Doyle [15] evaluated differences in patient ratings of the working alliance between a small sample (N = 15) of online therapy patients and normative data from a comparable face-to-face counselling sample. The authors found comparable evaluations of the working alliance in both samples. To gain a better understanding of the process and the mechanisms of change in online therapy we conducted a randomized controlled treatment study where the quality of the therapeutic alliance was systematically evaluated. Consequently, this investigation had several purposes.

First of all we aimed to examine if the approach introduced by Lange et al. [9] can be generalized to a sample from another country. According to the results of Lange et al. [9], we expected a significant statistical and clinical reduction of posttraumatic stress symptoms, depression and anxiety and other indications of psychopathology in the treatment group. Furthermore, we hypothesized that treatment effects can be sustained during the 3 months follow-up period.

The second purpose of this study was to examine the quality of the working alliance, its development through the course of therapy, and whether it moderates the impact of the observed change in symptoms. In accordance with findings on online relationship formation, it was expected that the working alliance would improve during the therapeutic process. Based on prior face-to-face research, it was expected to find significant correlations between patients' ratings of the therapeutic alliance at the end of treatment and treatment outcome. Patients' satisfaction with the online therapeutic contact was explored as an additional indicator of the online therapeutic alliance.

## Method

### Experimental design and patient flow

Participants were recruited by means of radio and newspaper advertisements as well as advertisements posted on websites for different groups (e.g., crime victims, sexual abuse victims, bereaved parents). Recruitment was performed from May to November 2003. Treatment and 3-months follow-up on all participants were completed in May 2004. The treatment approach was approved by the Netherlands Health Care Inspectorate and all participants gave written informed consent. Potential patients browsed through the website, which provided information about a) posttraumatic stress reactions, b) the study and its inclusion criteria, c) the treatment, d) the therapists and supervisors, and e) other treatment alternatives. Applicants were sent screening questionnaires by e-mail. Those who passed the screening were randomly assigned to the cognitive behavioural Interapy treatment group or a waiting list control group (WLC). The waiting list group received treatment after the post-assessment to the Interapy treatment condition. Patients who were excluded from the study were provided with information on where they could receive treatment elsewhere. The treatment lasted five weeks. Assessments were completed at three times (pre, post, and three-months follow-up).

**Figure 1:**
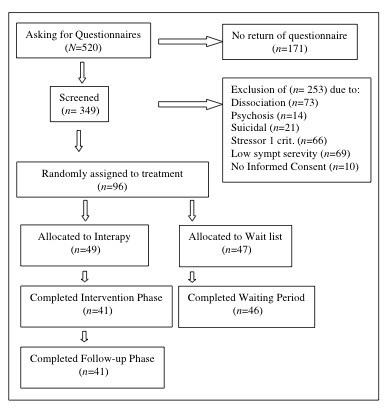
Flowchart showing progession of participants through the study.

In total, 520 people requested the questionnaires; 171 did not commit themselves to the screening process and 253 were excluded on the basis of the exclusion criteria (see below). Figure 1 summarizes the patient flow. Of the 96 patients who participated in this study 49 were randomly assigned to the treatment group and 47 to the WLC condition. Randomization was based on a computer generated randomization list.

### Participants

Participants were aged between 18 and 68 years, with an average age of 35 years; 90 % were female; 44% had a university degree, and a further 34% had a high school diploma (German Abitur). Forty two percent of the patients reported sudden or violent death of a close person and 32% reported sexual abuse, incest, or rape as traumatic event. On average, the traumatic event had occurred 8 years prior to the therapy (range 2–696 months). Scores on the IES-R indicated that the 96 participants suffered from high levels o distress. The mean scores on the intrusions (M = 23.1, SD = 7.1) and avoidance (M = 19.5, SD = 9.8) subscales were in the upper regions of the norm table for Dutch PTSD patients [16]. Neal et al. [17] found that an optimum cut-off score for the IES (which compromises the avoidance and intrusion subscales) of 35.0 produced the highest predictive value. Of the 96 participants 70% (n = 67) scored above this cut-off. The lowest IES scores in the sample were 20.0 indicating that all participants had at least a subsyndromal PTSD. Table 1 summarizes descriptive statistics on these demographic characteristics for participants of each group. Of the treatment group eight participants (16%) and of the waiting list control group one participant (2%) did not complete the second assessment. Most frequent reported reasons for dropping out were technical problems (network and computer) and emotional distress due to the writing about their stressful events.

**Table 1:** Demographic characteristics and type of trauma of treatment and waiting list group

	**Treatment group (N = 49)**		**Control group (N = 47)**	
	**Mean**	**SD**	**Mean**	**SD**
**Age (y)**	34	11.5	36	9.6
**Time since Trauma (y)**	10.7	.60	10.3	.51
	**N**	**%**	**N**	**%**
**Female sex**	41	84	45	96
**Marital status**				
Single	21	43	18	38
Partnership	25	51	18	38
**Education**				
High school (Abitur)	12	24	21	45
University	26	53	16	35
**Trauma**				
Sexual abuse/Rape	20	39	11	23
Death of close person	18	37	22	47
Accident	-	-	6	13
Physical disease	4	8	5	11

### Therapists

Two therapists conducted the treatment. Both were female trained clinical psychologists at the doctoral level who had received special training in the application of cognitive behavioural writing assignments for the treatment of PTSD. Their average age was 33 years. The 7-days training was provided by clinical psychologists of Interapy. The therapists participated in weekly supervision sessions.

### Assessment

To be included in the study participants had to: 1) have experienced a traumatic event that occurred at least one month prior to treatment and that met the criteria specified in DSM-IV [18], 2) be 18 years or older, 3) be fluent in written German, and 4) not be receiving treatment elsewhere. Online diagnostic self-report questionnaires were used to determine whether or not applicants were admitted to the program.

### Exclusion criteria

#### Severely depressed mood or suicidal intentions

Applicants were excluded if their score on the SCL-90 (Brief Symptom Inventory, BSI)[19] exceeded the cut-off score for the highly depressed group. Risk of suicide was measured using the Suicide Risk Assessment (SRT) [unpublished manuscript, University of Amsterdam, 2000], a six-item self-report questionnaire designed to capture suicidal tendencies. The assessment was conducted through the telephone as soon as a person indicated on the BSI that he/she suffered from suicidal ideations. It consists of questions tapping suicidal plans, previous suicide attempts, and current suicidal intentions.

#### Dissociative tendency

Dissociative symptoms were tapped using the Somatoform Dissociation Questionnaire (SDQ-5) [20]. The scale consists of five items, which are rated on a 5-point Likert scale (1 = not at all, 5 = very often). The internal consistency of the SDQ-5 is good (α = .80). Participants who scored above the cut-off score on the SDQ-5 were excluded from the treatment.

#### Risk of psychosis

Risk of psychosis was measured using the Dutch Screening Device for Psychotic Disorder [21]. This seven-item inventory has high internal consistency (α = .82) and is a good predictor of psychotic episodes. In a Dutch study, a high level of agreement was found between the self-reports of 33 patients and their clinicians' reports on them (α = .85) [21]. Since no German norm group exists as yet, the data from the Dutch norm group were used. Participants were excluded if they scored above the cut-off score. Participants were also excluded if they indicated the use of neuroleptics.

#### Alcohol and drug abuse

To gather miscellaneous information, including drug and alcohol consumption in terms of quality (amount and sort of alcohol/drug) and frequency of consumption, medications as well as degree of computer and Internet experience a short biographical checklist was administered. Participants were excluded if they indicated heavy alcohol or drug abuse.

### Outcome measures

#### Posttraumatic stress

The revised version of the Impact of Event Scale (IES-R) [22] was used to assess symptoms of posttraumatic stress. The scale consists of 22 items constituting the subscales 1) intrusions, 2) avoidance, and 3) hyperarousal, the three main characteristics of psychological dysfunction after a traumatic life event. Participants were asked to indicate the frequency of each symptom over the past 7 days on a 4-point Likert scale (0,1,3,5).

#### Depression and anxiety

The depression and anxiety subscales of the short form of the SCL-90 (Brief Symptom Inventory, BSI) [19] were used to measure the effects of treatment on psychological dysfunction in dimensions related to symptoms of posttraumatic stress. The two subscales consist of six items each. Each item is rated on a 5-point Likert scale (0 = not at all, 4 = extremely).

#### Mental and physical health

Physical and psychological functioning was measured using the 12 item version of the medical Outcome Study Self-report Form (SF-12) [23].

#### Quality of the therapeutic alliance

##### Working alliance

The Working Alliance Inventory (WAI) [24] was designed to apply to diverse therapeutic orientations and modalities. The WAI assesses three primary components of the working alliance: 1) agreement between patient and therapist on the therapeutic tasks ('agreement on therapeutic tasks' subscale: reliability coefficient in this study: α = .73), 2) agreement between patient and therapist on the therapeutic goals ('agreement on therapeutic goals' subscale: reliability coefficient in this study: α = .80), 3) the degree of mutual trust, acceptance, and confidence between client and therapist ('therapeutic bond' subscale: reliability coefficient in this study: α = .79). The composite score (reliability coefficient in this study: α = .88) is used as a global measurement of working alliance. In this study, the short version of the instrument (WAI-S) [25] was used. Busseri and Tyler [26] have shown that the two versions correlate highly in terms of their psychometric and predictive qualities, and are thus interchangeable. Respondents were asked to rate each statement on a 7-point Likert scale ranging from 1 (never) to 7 (always). Two versions of the WAI-S are available: a client version and a therapist version. Both versions were used in this study.

##### Internet-specific questions

In addition to the WAI, questions concerning patients' satisfaction with the Internet-based contact were asked (e.g. How did you experience the fact being treated through the Internet instead of face-to-face?).

##### Treatment Protocol

Patients were allocated to two weekly 45-minute writing assignments over a five-week period (10 essays in total). The therapy consisted of three treatment phases: 1) self-confrontation, 2) cognitive reconstruction, and 3) social sharing. After the fourth writing session, which constituted the end of the first treatment phase, the Working Alliance Questionnaire was administered. The treatment procedure is described in detail by Lange, Schoutrop, Schrieken, and van de Ven [27] and will only be outlined in brief here.

#### First Phase: self confrontation

At the beginning of the treatment, participants received psycho-education about the mechanisms of exposure. In the first phase, the therapists helped the patients to focus on the most painful images and thoughts and encouraged the patient to write about them. The patients were instructed to describe the traumatic event thoroughly including their intimate fears and thoughts concerning the traumatic experience. To increase the effect of the exposure, patients were asked to write in the first person and in present tense and to give detailed descriptions of all sensory details they had experienced during the traumatic event including olfactory, visual and auditory stimuli. Participants were explicitly asked not to concentrate on style, grammar, spelling, or the chronological order of their essays. The therapists checked whether patients explicitly addressed the traumatic event as described above. If needed the therapist supported the patient to address the avoided features more forcefully. The following is an example of a writing assignment for essays 3 and 4:

"For the next two texts, I would like to ask you to choose one moment of your traumatic event. One moment that you can hardly bear to think about, but that keeps intruding on your thoughts. Write down the most painful memories and emotions you have when you think about it and describe everything that you experience – every feeling, every thought and physical reaction."

#### Second phase: Cognitive restructuring

During the second phase, patients received psycho-education about the principles of cognitive restructuring. The goal of this phase was to form a new perspective on the traumatic event and to regain a sense of control. Participants wrote a supportive letter to an imaginary friend who had been through the same experience. In this letter, the patient was instructed to reflect on the addressee's feelings of guilt and shame, challenge dysfunctional automatic thinking and behaviour patterns, and correct unrealistic assumptions. Furthermore, patients were encouraged to consider potentially positive consequences of the traumatic event for that person's life and the lessons to be learned from it. An example of an instruction for the first two essays in the second phase is as follows:

"Imagine you are writing a supportive letter to your friend Hanna, who experienced the same situation as you. Could she have foreseen what happened? Do you think she was responsible for this?"

#### Third Phase: Social sharing and farewell ritual

During the third phase, patients received psycho-education about the positive effects of social sharing. In a final letter, they then took symbolic leave of the traumatic event. Patients summarize what has happened to them, reflect on the therapeutic process and describe how they are going to cope now and in the future. Patients could address the letter either to themselves, to a close friend, or another significant person involved in the traumatic event. The letter did not ultimately have to be sent.

"You wrote that you would like to address the letter to your mother. First, I would like to ask you to describe the circumstances of what happened. Which moments were so important that you would like to tell her about them? What meaning does this experience have in your life. What plans do you have for the future? Who is important in your life and who can support you in the future? It is important to give the past, the present and the future the same weight in this letter."

At the beginning of each writing phase, patients proposed individual timetables as to when they planned to write. Halfway through and at the end of each treatment phase, patients received feedback and further writing instructions, which were based on the treatment manual but tailored to patients' specific needs. Important aspects of this feedback were recognition and reinforcement of the patients' independent work, positive feedback and motivation, as well as frequent summaries and encouraging patients to voice questions and doubts.

### Data Analysis

Descriptive statistics was used to examine the demographic data. Chi-square analyses were conducted to determine differences between two groups in terms of gender, education level, or marital status. Independent samples T-tests were used to assess differences in the mean age, years since the trauma and pre-treatment psychopathology. Repeated measures analysis of variance (MANOVA) was used to analyze treatment effects, with two groups (intervention and control) and two time points (pre-test and follow-up). The principal interest was in the group × time interaction effect. The analysis was carried out according to intention-to-treat principles, so that all persons who completed a pre-test questionnaire were included, even if they subsequently dropped out. In such cases, the pre-test score was substituted for the missing value, so that no improvement was assumed. Effect sizes were calculated using Cohen's *d *for repeated measures [28] to quantify the magnitude of change in mean symptoms between pre- and post-test and between pre-test and 3-months follow-up, respectively. By Cohen's standards for research in the behavioural sciences, an effect size d = .80 for treatment effects in psychotherapy is considered large. In addition to examining statistical significance, we were also interested in whether symptom changes were clinically meaningful. To assess the clinical significance of changes due to treatment, the proportion of individuals who returned to a normative level of functioning (change of diagnostic category) on the main dependent variable of interest was computed [29]. The data were analyzed with 2 × 2 chi squares comparing the two groups on whether the participants who initially met criteria for PTSD continued to meet it at post-treatment or not. To determine the relationship between patients' scores on the Working Alliance Inventory and post-treatment scores partial correlations after partialling initial symptom levels for post-treatment scores were calculated. To estimate the variance accounted for therapeutic relationship on the main outcome variable (IES-R) multiple regression analyses were used to further explore possible mediator or suppressor effects of the patients' ratings of the working alliance.

## Results

Chi-square analyses failed to reveal any significant differences between the two conditions in terms of gender, education level, or marital status, and *t *tests showed no significant differences in terms of age, years since the trauma or pre-treatment psychopathology.

### Treatment effects

The means and standard deviations for intrusions, avoidance, hyperarousal, depression, anxiety, mental health and physical functioning of each group at the different assessment periods are presented in Table 2. Also in this table are values for the Groups × Time interaction from the Groups × Times repeated measures MANOVA and whether group change is significant from pre-treatment to post-treatment (and from post-treatment to 3-months follow-up). Table 2 shows significant changes on all measures (except the physical functioning scale of the SF-12) from pre-treatment to post-treatment for those receiving the Interapy treatment. The three months follow-up revealed further arithmetic improvement from post-test to follow-up in the treatment group on all measures except the IES-R intrusion subscale. However, none of these changes were significant. As demonstrated in Table 2, also individuals in the waiting list control condition experienced a slight but significant improvement on trauma-related symptoms (intrusions, avoidance, hyperarousal) and depression.

**Table 2:** Psychological test results for the treatment group (Interapy) and the waiting list control group (WLC) at pre-treatment and post-treatment and 3-months follow-up: Intention-to-Treat Analysis.

									**Groups × Pre-Post Effect**	
	Pre-test		Post-test		Follow-up		Effectsize Pre to Post	Effectsize Pre to 3-months	***F***	***p***
	M	SD	M	SD	M	SD				

**Intrusions IES-R**										
**Treatment**	23.0^a^	(6.4)	12.3^b^	(8.7)	12.7^b^	(8.1)	1.40	1.41	*F *= 21.52	*p *< .001
**Control**	23.3^a^	(7.89)	20.7^b^	(9.2)	-	-	0.30			
**Aoidance IES-R**										
**Treatment**	19.9^a^	(9.8)	10.1^b^	(10.2)	9.7^b^	(9.9)	.98	1.0	*F *= 10.00	*p *< .005
**Control**	19.0^a^	(10.0)	16.0^b^	(10.5)	-	-	0.29			
**Hyperarousal IES-R**										
**Treatment**	22.1^a^	(6.5)	11.0^b^	(9.0)	10.0^b^	(8.5)	1.41	1.60	*F *= 25.49	*p *< .001
**Control**	19.1^a^	(9.5)	16.5^b^	(9.9)			0.27			
**Depression BSI**										
**Treatment**	10.1^a^	(4.0)	5.3^b^	(4.3)	4.9^b^	(4.2)	1.16	1.27	*F *= 7.38	*p *< .05
**Control**	9.4^a^	4.7	7.2^b^	4.9			.46			
**Anxiety BSI**										
**Treatment**	9.1^a^	(3.4)	5.2^b^	(3.8)	4.7^b^	(3.8)	1.08	1.22	*F *= 10.73	*p *< .001
**Control**	7.5^a^	(4.7)	6.5^a^	(4.7)			.21			
**Mental Health SF-12**										
**Treatment**	34.6^a^	(5.6)	39.7^b^	(7.4)	40.0^b^	(7.6)	.77	.80	*F *= 5.95	*p *< .05
**Control**	35.5^a^	(6.5)	36.9^a^	(6.2)			.22			
**Physical Health SF-12**										
**Treatment**	46.7^a^	(5.2)	47.2^a^	(5.2 =	47.9^a^	(5.0)	.10	.24	*F *= .001	n.s.
**Control**	46.0^a^	(5.1)	46.6^a^	(5.2)			.17			

^a,b ^Means within a column which share a superscripts do not differ at p = 0.05 Note: Treatment group: *n *= 49, control group: *n *= 46

### Effect Sizes

For PTSD symptoms (intrusions, avoidance, hyperarousal), large effect sizes of treatment were found at post-treatment (d = .98 to d = 1.41) and 3-months follow-up (d = 1.0 to d = 1.60). At post-treatment, large treatment effect sizes were also found for symptoms of depression (d = 1.16) and for anxiety (d = 1.08) and mental health (d = .77), but the treatment effect sizes for physical functioning were near zero at post-treatment (d = .10).

### Clinical Significance

As there is no internationally used cut-off for the IES-R available the categorization is based on the IES including the subscales avoidance and intrusions with a cut-off of 35.0 [17]. The analysis revealed that the treatment group was significantly superior to waiting list (χ^2 ^= 9.29, df = 1, p = .002). In summary, 74% of those with initial PTSD treated by Interapy had thus changed diagnostic category, compared to 21% of those on the waiting list who were assessed twice.

### The Working Alliance

It was expected that the working alliance would improve during the therapeutic process. In addition, it was hypothesized to find a significant correlation between patients' alliance ratings at the end of treatment and treatment outcome. With regard to the development of the online therapeutic alliance it was found that patients' ratings of the working alliance significantly improved during treatment (F 1,40) = 25.45, p < .001). As shown in Table 3 there was no significant change in alliance ratings of the therapists. Post-treatment scores were correlated with patients' and therapists' ratings of the working alliance at the end of treatment. Table 3 shows partial correlations between the subscales and the composite scores of the patients' scores on the Working Alliance Inventory and the post-treatment scores after partialling initial symptom levels for post-treatment scores. Also shown in Table 3 are inter-correlations of the patient version of the WAI and correlations with the composite score of the therapists' ratings of the alliance.

**Table 3:** Characteristics of the patients' Working Alliance Inventory (WAI-P) and correlations with therapist composite ratings (WAI-T) and psychopathology in the treatment group (*n *= 41).

		**Time of assessment**		**Intercorrelations of the WAI Correlation of 10^th ^session data**									
**Working Alliance Invent. (scale from 1–7)**		4^th ^M (SD)	10^th ^M (SD)	Test *p*	2	2a	2b	2c	IES	BSI depr.	BSI anx.	SF12^1 ^Psych.	SF12^1 ^Physic

1	Therapists view therap. alliance (composite)	5.6 (.72)	5.8 (.98)	n.s.	.37*	.21	.52**	.17	-.30	-.46*	-.33*	.36*	.11
2	Patients view therap. alliance (composite)	5.8 (.64)	6.3 (.54)	> .001		.92**	.87**	.77**	-.50*	-.50*	-.50*	.35*	.20
2a	Agreement on therapeutic goals	5.8 (.77)	6.3 (.65)	> .005			.85**	.52**	-.53**	-.52*	-.40*	.40*	.13
2b	Agreement on therapeutic tasks	5.7 (.83)	6.2 (.69)	> .001				39*	-.53**	-.61**	-.38*	.48*	.10
2c	Therapeutic bond	6.2 (.69)	6.4 (.57)	> .05					-.25	-.17	-.48*	.03	.28

* *p *< .05; ** *p *< .001 ^1 ^reversely coded

At the end of treatment significant inverse correlations could be observed between the all subscales of the patients' alliance ratings and all psychological outcome measures (the SF-12 mental health is scored reversely thus a positive correlation was found in this case). The more positive patients experienced the therapeutic relationship at the end of treatment the less psychological symptoms they reported after the treatment. No significant correlation was found between physical function and alliance ratings. Composite scores of therapists' alliance ratings were significant negatively related to anxiety, depression and the SF-12 mental health subscale.

To estimate the variance accounted for therapeutic relationship on the main outcome variable (IES-R) multiple regression analyses were used to further explore possible mediator or suppressor effects of the patients' ratings of the working alliance. The pre-treatment scores on the IES-R were entered as the first independent variable to control for pre-treatment level of trauma symptoms. Results revealed that the working alliance rated by patients measured at the end of therapy predicted 15% of the variance in the post-treatment scores of the IES-R (adjusted R-square = .148; F2,39 = 8.15, p < .05). Participants who had a better therapeutic relationship post-treatment benefited more from treatment.

### Internet-specific aspects of the therapeutic alliance

After finishing the treatment patients were asked how they experienced the fact being treated through the Internet (see Table 4). Eighty-six percent of the patients described the therapeutic contact as personal, 76% reported positive attitudes to being treated through the Internet instead of via face-to-face and 60% of the patients did not miss the face-to-face communication with a therapist.

**Table 4:** Satisfaction with the online therapeutic contact (*N *= 41)

**Questions**	**Answers**	**Percentage**
Did you miss face-to-face communication with your therapist for example with regard to support and instructions?	No	60%
	Yes	17%
	I don't know	12%
How did you experience the fact being treated through the Internet instead of face-to-face?	Pleasant	76%
	Unpleasant	5%
	I don't know	19%
What was the contact between you and your therapist like?	Personal	86%
	Impersonal	2%
	I don't know	12%

## Discussion and Conclusion

The research questions we investigated in this study were twofold. Our first hypothesis addressed the overall impact of an Internet based cognitive behavioural intervention (Interapy) on a sample of patients with PTSD or subsyndromal PTSD. We found significant statistical and clinical effects that indicated symptom reduction of PTSD in the treatment group. Furthermore, a reduction in psychological symptoms related to depression, anxiety and mental health accompanied improvements in PTSD symptoms. However, the participants with trauma-related symptoms and depression in the control group also improved significantly on trauma-related symptoms and depression. Furthermore, results indicate that treatment gains were maintained up to 3 months after the completion of treatment. This is in line with previous studies of internet-driven CBT for posttraumatic stress reactions [9], complicated grief [30] and CBT interventions in face-to-face studies [10,31]. This was the first cross-culturally applied study examining Interapy in a German speaking sample. It replicated the findings of Lange et al. [9] and validates this treatment approach by indicating effectiveness, acceptability and the applicability across different countries. Although, several effective treatment approaches for PTSD have been available for a considerable time, accessibility remains a problem due to difficulties in establishing and maintaining effective methods of dissemination of these treatment methods to treatment providers [32]. In the Netherlands, Interapy is already integrated into the regular health care system and is accessible nationwide. But since the assessment is exclusively based on questionnaires no formal diagnosis has been able to be established over the Internet. In face-to-face interactions the assessment is carried out by trained psychologists during an interactive diagnostic process. Assessment models should be developed to be implemented over the Internet. Thus, further evidence is needed before conclusions about the generalizability for a general population of PTSD patients can be drawn. Future research should directly compare face-to-face with Internet based intervention after establishing a clinical diagnosis face-to-face to be able to evaluate the efficacy of Internet based therapy more clearly.

Furthermore, we were interested in finding out whether a positive and stable relationship can be maintained online, whether the therapeutic alliance would improve throughout treatment and whether the quality of the online therapeutic relationship would have a moderating effect on treatment outcome. High ratings of the working alliance (at the end of treatment: patients M = 6.3; therapists M = 5.8 on a scale from 1–7) of both parties were obtained. Callahan, Price, and Hilsenroth [33] assessed the working alliance in face-to-face therapy with the WAI at the end of treatment. They found mean alliance ratings of M = 5.5 (child abuse survivors) and M = 5.4 for patients with other psychiatric disorders. Surprisingly, the bond-dimension of the working alliance which comprised statements such as: "Me and my therapist trust each other" was rated particularly high in our study even at an early stage of treatment (4th session). Also, a relatively low drop-out rate (16%) and the fact that the majority rated this exclusively internet-based contact as positive (76%) and personal (86%) indicated stable and positive therapeutic relationship online. Significant improvement of the therapeutic relationship rated by patients could be observed during the course of treatment. Findings on face-to-face studies identified three typical patterns: a stable alliance pattern, a linear growth pattern and a u-shaped pattern [34]. Possibly, the alliance formation observed in this study is similar to the development of the therapeutic relationship in face-to-face therapies. Alternatively, it might also be the case that the therapeutic alliance online, particularly in the eyes of the patients, may not have stabilized by the fourth writing session. This would be in line with Walther [6] who found that the difference in quality between online and face-to-face relationships is moderated by the duration of the relationship and the frequency of contact. In other words, the degree of intimacy is influenced by the amount of information that is exchanged. Repeated assessment of the working alliance and an immediate comparison with a face-to-face intervention would be needed to find out whether this would also apply to online therapeutic relationships. Therapists' alliance rating showed no variation.

According to our hypothesis we found a substantial correlation between the late therapeutic alliance and treatment outcome. This is in line with previous findings of face-to-face studies of CBT showing that substantial amounts of outcome variance were uniquely accounted for by alliance scores [35]. However, an alternative explanation for the correlation between working alliance and treatment outcome might be that ratings of the quality of the working alliance might have been confounded with outcome. Thus, instead of being a predictor for outcome the rating of the alliance would be an additional indirect measure of outcome. Previous analysis of the online working alliance early in treatment revealed no substantial correlation between the working alliance and treatment outcome [36]. Further research is needed to understand the therapeutic contribution of the online therapeutic alliance. Measurement of the working alliance and symptom level at several points during the whole therapeutic process would help to understand the relation between online therapeutic alliance and outcome.

In the current study, we sought to ascertain the efficacy of an internet-driven treatment for PTSD and the quality and the role played by the online therapeutic alliance. The examination of an online therapeutic alliance is of particular relevance since it has proven to be a stable predictor in face-to-face therapy.

Among the limitations of this study is the screening strategy for the recruitment of the patients. We deliberately handled strict exclusion criteria for participation in this study. We excluded 72% (n = 253) of the patients who wanted treatment but did not meet the inclusion criteria. This might limit the generalizability of our results. Also, the sample was mainly female, better educated and younger than the general population. Another methodological concern might be the choice of the questionnaire. We used the frequently applied Working Alliance Inventory because of its pantheoretical nature which allowed its use in many different treatment approaches. However, the WAI was not designed for an internet-driven type of therapy and it might be that it is a less valid instrument for capturing an online therapeutic alliance. A further limitation is that we included a waiting list control group instead of placebo control group. This design will likely result in higher effect sizes compared to a placebo control group. In addition, as we employed a waiting list controlled design, it would have been unethical to deny treatment to those patients originally randomized to the waiting list. Consequently, there is no control group against which the outcomes at the follow up assessments of the treated sample can be compared. This limits the evaluation of long-term effects of this intervention. Finally, treatment outcomes were measured mainly by self-rated questionnaires administered through the Internet only. Interviews or other independent assessments would have added to the validity and clinical value of the results.

Although the results of the present study are promising, there is a need for further studies concerning the applicability and efficacy of online therapy and specific underlying processes such as the development of the therapeutic alliance and its distinctive cross-method features. Further analysis of the 18 months follow-up data and the examination of other potentially relevant moderators such as posttraumatic growth [Maercker & Knaevelsrud, in preparation] will hopefully enhance our understanding of online therapeutic processes. Considering that online therapy is gaining acceptance [37] and provides a cost-efficient, worldwide accessible alternative it is imperative that we increase our understanding of this new treatment approach.

## Abbreviations

**BSI**: Brief Symptom Inventory

**CBT**: cognitive-behavioral therapy

**DSM-IV**: Diagnostic and Statistical Manual of Mental Disorders, Fourth Edition

**IES-R**: revised version of the Impact of Event Scale

**PTSD**: posttraumatic stress disorder

**SCL-90**: Symptom Checklist

**SDQ-5**: Somatoform Dissociation Questionnaire

**SRA**: Suicide Risk Assessment

**WAI:**Working Alliance Inventory

**WAI-S**: short version of the WAI

## Competing interests

The author(s) declare that they have no competing interests.

## Authors' contributions

CK has made substantial contributions to conception and design, coordination, acquisition of data, analysis and interpretation of data and writing the paper. AM has made substantial contributions to conception and design, analysis and interpretation of data and manuscript revising. Both authors read and approved the final manuscript.

## Pre-publication history

The pre-publication history for this paper can be accessed here:


